# The Rise of CRISPR/Cas for Genome Editing in Stem Cells

**DOI:** 10.1155/2016/8140168

**Published:** 2016-01-06

**Authors:** Bing Shui, Liz Hernandez Matias, Yi Guo, Ying Peng

**Affiliations:** ^1^Department of Biology, Carleton College, Northfield, MN 55057, USA; ^2^Department of Biology, University of Puerto Rico, Rio Piedras, San Juan, PR 00931, USA; ^3^Department of Biochemistry and Molecular Biology, Mayo Clinic, Rochester, MN 55905, USA; ^4^Division of Gastroenterology and Hepatology, Mayo Clinic, Rochester, MN 55905, USA

## Abstract

Genetic manipulation is a powerful tool to establish the causal relationship between a genetic lesion and a particular pathological phenotype. The rise of CRISPR/Cas9 genome-engineering tools overcame the traditional technical bottleneck for routine site-specific genetic manipulation in cells. To create the perfect* in vitro* cell model, there is significant interest from the stem cell research community to adopt this fast evolving technology. This review addresses this need directly by providing both the up-to-date biochemical rationale of CRISPR-mediated genome engineering and detailed practical guidelines for the design and execution of CRISPR experiments in cell models. Ultimately, this review will serve as a timely and comprehensive guide for this fast developing technology.

## 1. Introduction

Genome-engineering tools facilitate site-specific DNA deletions, insertions, inversions, and replacements. These manipulations of the complex eukaryotic genome help researchers understand the function of genes in a given cellular context, explore the mode of gene regulation at the endogenous locus, and, most importantly, model human disease conditions using* in vitro* cellular models or* in vivo* model organisms.

Since the emergence of designer nucleases based on DNA base recognition by modular protein motifs, such as Zinc Fingers in Zinc Finger Nucleases (ZFNs) [1–3], as well as TALE domains in transcription activator-like effector nucleases (TALENs) [4, 5], site-specific DNA manipulations in eukaryotic cells have passed a critical efficiency and specificity threshold to enable routine applications in a laboratory. The recently developed, explosively popular CRISPR/Cas9 (clustered regularly interspaced palindromic repeats/CRISPR-associated) genome-engineering system has transformed discovery in this exciting era. CRISPR/Cas was first discovered in prokaryote adaptive immunity [6–8] and has now been more extensively adapted for eukaryotic genome engineering than ZFNs and TALENs [9]. The most widely utilized class, the type II CRISPR/Cas9 system from* Streptococcus pyogenes*, offers users the greatest ease and modularity for design and execution of any genome-engineering experiments [10–13]. However, limitations and common practical pitfalls of the CRISPR/Cas9 system have not been sufficiently and systematically summarized and emphasized for the emerging population of potential users, in large part due to the great enthusiasm accompanying the system's amazing rise in popularity.

In this review, practical issues associated with the design and execution of a typical CRISPR experiment will be discussed, especially in the context of modeling human diseases using stem cells. Due to the limitation of the current scope, this paper will discuss neither earlier designer nucleases (ZFNs and TALENs) nor applications of CRISPR on model organisms, although similar rationale and general principles discussed in the following sections would also apply to these applications.

## 2. The Discovery of CRISPR/Cas System

The CRISPR system was first discovered in bacteria as an “adaptive immune system” against plasmids, viral DNA, or RNA [6–8]. This “memory system” can destroy DNA or RNA if reinfection occurs in the same bacteria or in its descendants [14–19]. Three types of CRISPR loci exist, all of which acquire short pieces of DNA called spacers from foreign DNA elements [20]. Spacers are integrated into the bacterial genome during the process of CRISPR adaptation. They are usually inserted into the CRISPR locus that contains short, partially palindromic DNA repeats to form loci that alternate repeated elements (CRISPR repeats). These loci are subsequently transcribed and processed into small interfering RNA that guides nucleases for sequence-specific cleavage of complementary sequences. Through these stepwise but continuous evolutions of adaptation, CRISPR repeat RNA (crRNA) biogenesis and foreign DNA targeting generated sophisticated CRISPR-based adaptive immune systems in nearly half of the bacterial species, as well as in most archaea [21].

The sequence in the exogenous nucleic acid element corresponding to a CRISPR spacer was defined as a protospacer [22]. For proper targeting by type I and II CRISPR systems, the protospacer is usually flanked by a system-specific, highly conserved CRISPR motif, namely, a protospacer adjacent motif (PAM) [23]. Most PAMs are typically 2 to 5 highly conserved nucleotides, either on the 5′ end of protospacer (type I system) or on the 3′ side (most type II systems). A significant feature of the PAM for the CRISPR system is to distinguish the foreign DNA against the host genome; thus, only the PAM-bearing invading sequence will be targeted for destruction.

## 3. Different Classes of CRISPR/Cas

Among the three different types of CRISPR loci, type I and III loci involve a complex panel of multiple Cas proteins that form ribonucleoprotein (RNP) complexes with CRISPR RNA to target foreign sequences [15]. However, the type II CRISPR system uses a much smaller number of Cas proteins to perform this core function. Type II CRISPR loci have three subdivisions. The most commonly used CRISPR system for eukaryotic genome engineering is adopted from a type II A system from* S. pyogenes*, where a single Cas9 protein (spCas9) is responsible for both forming the CRISPR-RNP complex and subsequent DNA cleavage. For the practical reason of simplicity, most genome-engineering applications use one hybrid RNA (guide RNA, gRNA) combining the essential structural features of the transactivating RNA (tracrRNA) and crRNA duplex [10]. The single-chain gRNA is used here in subsequent discussions.

Besides spCas9, a few other orthologous Cas9 proteins from similar type II CRISPR systems share the core feature as the sole protein component for RNA-guided targeting. The Cas9 proteins of* Streptococcus thermophilus*,* Neisseria meningitidis*, and* Treponema denticola* demonstrated comparable genome-editing efficiency to spCas9 (Table 1) [24–27]. These Cas9 proteins have different sizes, mostly due to their target recognition domains (REC) [28]. Significantly, orthologous Cas9 proteins differ in the specific PAM sequences used for targeting; thus, they can be used in the same cell when paired with their corresponding crRNA to recognize their corresponding targets without interfering with each other [29–31]. This characteristic enables sequence flexibility of CRISPR experiments by offering a variety of Cas9 proteins to target virtually any particular sequence [25]. This orthogonality was best demonstrated by recent work that allowed the labeling of distinct genomic regions using different inactivated Cas9-fluorescent fusion proteins simultaneously in a single live cell [32, 33]. Although most Cas9 proteins from type II CRISPR system have one or more optimal PAMs, there is also considerable flexibility in terms of PAM recognition. For example, spCas9 recognizes NGG as its optimal PAM sequence, while NAG can also be recognized with lower frequency ([12] and subsequent). This plasticity might arise from continuous selection pressure on bacterium to target evolving viral sequences [34]. In practice, this plasticity poses considerable challenges due to the off-targeted recognition of alternative PAM sequences [12]. On the other hand, this flexibility allows further engineering of different Cas9 proteins to optimize or modify PAM preference. Initial progress has been made toward generation of spCas9 with more rigid NGG PAM recognition and modification of the PAM preferences [35]. In a few years further biochemical characterization of native orthogonal Cas9 proteins with their PAM preferences and protein engineering efforts on characterized Cas9 proteins will likely generate a full repertoire of Cas9 proteins with high specificity covering virtually any 2~5-nucleotide PAMs.

A recent important addition to the CRISPR toolbox is the characterization of Cpf1, a class II CRISPR effector that is distinct from Cas9. Cpf1 is a single RNA-guided endonuclease that uses T-rich PAMs and generates staggered DNA double-stranded breaks instead of blunt ends [36]. Its smaller protein size and single RNA guide requirement may make future CRISPR applications simpler and with more precise control.

## 4. Cas9 Enzymology

**Figure 1:**
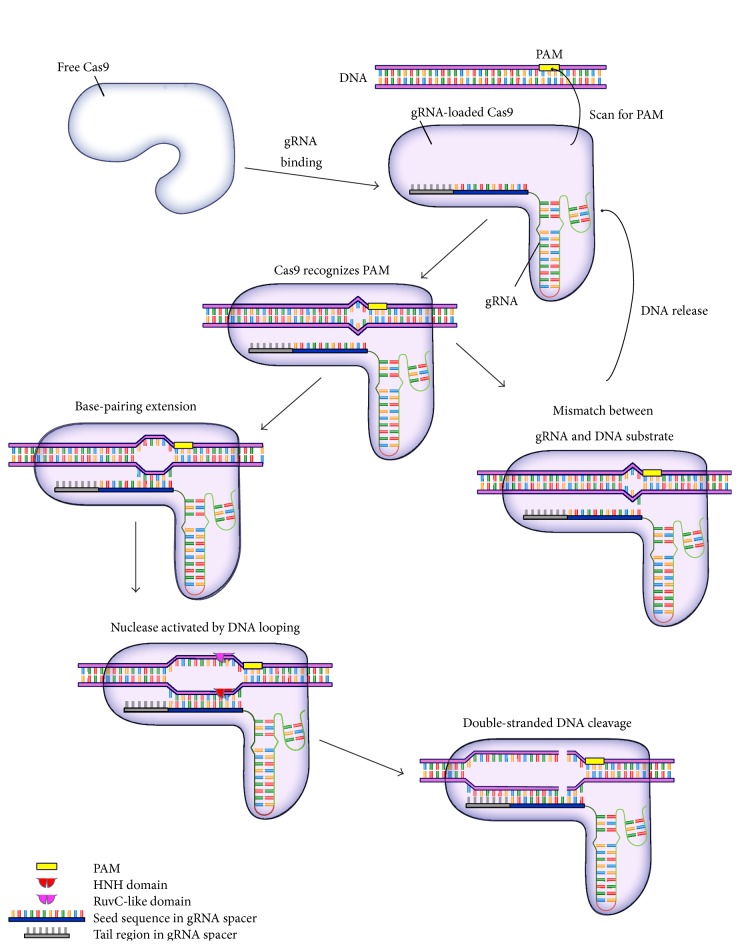
A proposed model for Cas9 endonuclease to trigger DNA cleavage. A conformational change is induced once the Cas9 protein binds to gRNA, allowing it to search for the DNA substrate. The REC lobe of Cas9 scans for the PAM in the genome. PAM recognition helps local unwinding of dsDNA 5′ to the PAM region. The unwound DNA is transiently stabilized by protein/ssDNA interaction. Successful base pairing between the ssDNA portion and the gRNA further extends the ssDNA loop. A critical loop size may trigger the enzymatic activity of Cas9 to make the double-stranded cut. Afterwards, Cas9 remains bound to the DNA substrate. If the base pairing between ssDNA and gRNA is blocked by mismatches, the ssDNA loop collapses to release the Cas9 protein.

The Cas9 protein contains two independent endonuclease domains: one is homologous to the HNH endonuclease and the other one to the RuvC endonuclease (Figure 1) [10]. Each domain cleaves one strand of double-stranded DNA (dsDNA) at the target recognition site: the HNH domain cleaves the complementary DNA strand (the strand forming the duplex with gRNA), and the RuvC-like domain cleaves the noncomplementary DNA strand [10]. Recent CRISPR/Cas9 complex structural analysis [37, 38] revealed a two-lobed structure for Cas9: a recognition (REC) lobe and a nuclease (NUC) lobe. Cas9 interacts with the RNA-DNA duplex using the REC lobe in a largely sequence-independent manner, implying that the Cas9 protein itself does not confer significant target sequence preference. One caveat of the CRISPR/Cas9 system is that gRNA-loaded Cas9 endonuclease cleavage is not completely dependent on a linear guide sequence, since some off-target sequences were shown to be cut with similar or even higher efficiency than the designed target sites [12, 39–42]. In general, mismatches between the first 12 nucleotides (nts) of the gRNA (seed sequence in gRNA spacer, Figure 1) and the DNA target are not well tolerated, suggesting high sequence specificity in the PAM-proximal region. However, mismatches beyond the first 12 nts can be compatible with efficient cleavage (tail region in gRNA spacer, Figure 1) [12]. Structural biology insights into the Cas9-gRNA RNP complex revealed that the 12-nt sequence is in a fixed “seed” configuration even prior to the DNA substrate binding, whereas the 5′ end of gRNA remains unstructured. While generally true, it is an oversimplification, and the sequence recognition specificity of the CRISPR system is a topic of active investigation [39–44]. Notably, shorter gRNA with up to a 5000-fold reduction in off-target effects was recently described [45]. Adding two additional Guanine (G) nucleotides at the 5′ end of gRNA in some circumstances modestly improves the specificity of the CRISPR/Cas9 system [46], possibly by altering gRNA stability, concentration, or secondary structure. The relaxation of sequence specificity of the RNA-guided endonuclease system remains the biggest challenge for its usage in genome engineering. A recent biophysical study [37] for the thermodynamic properties of Cas9 binding provided a likely explanation for the features of specificity outlined above, and further analyses along these lines will be valuable to further refine design guidelines.

A degree of structural flexibility was found from the DNA-gRNA duplex-loaded Cas9 crystallography structure [38], which was substantiated by an independent crystallography and single-particle electron microscopy study on both* S. pyogenes* and* A. naeslundii* Cas9 [37]. This study demonstrated that a conformational rearrangement is induced by gRNA binding to Cas9, shaping a central channel to accommodate the DNA substrate (Figure 1, gRNA binding) [37]. Detailed structural information is lacking for how Cas9 recognizes targeted sequences within the genome and triggers the specific DNA cleavage after sequence recognition. However, the RNA-loaded Cas9 protein reads the PAM in its base-paired configuration (Figure 1, scan for PAM). The recognition of dinucleotide GG in PAM simultaneously allows for the local stabilization of the unwound target DNA immediately upstream of the PAM sequence, which might compensate for the energy cost of local DNA strand separation starting immediately upstream of PAM (Figure 1, Cas9 recognizes PAM) [47]. A recent biophysics study for Cas9-mediated DNA recognition* in vitro* further revealed that Cas9 does not behave as a typical nuclease [48]. First, gRNA-loaded Cas9 enzymatic activity does not follow Michaelis-Menten kinetics, since Cas9 protein stably associates with target sites on DNA even after inducing a double-strand break. Thus, the key requirement for successful CRISPR-mediated genome engineering is efficient and precise target locating. Secondly, gRNA-loaded Cas9 finds the target sequence using 3D diffusion without obvious sliding on the DNA substrate. Cas9 pauses on DNA for interrogation once it recognizes a PAM sequence. Many of these reactions are transient and do not lead to DNA cleavage. In agreement with this “pausing” behavior of the gRNA-loaded Cas9 on the DNA substrate* in vitro*, this mode of transient DNA binding on a nonmatching target is stable enough in cells to be detected using genome-wide CHIP-Seq (Chromatin Immunoprecipitation Sequencing) [43]. Besides the highly enriched binding of Cas9 at its on-target site, numerous binding events with lower frequency can be observed around a short motif of 5~10 nucleotides matching the PAM-proximal region on a gRNA plus NGG PAM sequence [43]. Thus these “off-targeted” bindings likely involve partial base pairing between gRNA and the PAM-proximal sequence. Without intrinsic DNA helicase activity, how Cas9 facilitates the strand replacement on its DNA substrate by the gRNA is not known. It is suggested to be a thermodynamically favorable process upon PAM recognition, and the unwinding of local DNA base pairing was suggested to be in a directional and sequential manner, starting at the 3′ end of the target sequence adjacent to PAM and progressing in the 5′ direction of the DNA substrate (Figure 1, base-pairing extension) [47, 48]. The Cas9 protein likely stabilizes the locally unwound DNA, allowing further stabilization of the single-stranded DNA chain by continuous formation of Watson-Crick base pairing with the gRNA (Figure 1, base-pairing extension). If base pairing is blocked due to a mismatch between the DNA substrate and the gRNA, the thermodynamic energy of the DNA-Cas9 interaction might be insufficient to maintain a significant portion of unwound DNA. In this case, partially unwound DNA will return to its duplex state, and the DNA-Cas9 interaction will attenuate simultaneously (Figure 1, mismatch and DNA release). These observations provide an attractive stepwise substrate-unwinding model for target recognition and cleavage by the gRNA-loaded Cas9 protein. This model predicts that only perfectly or nearly perfectly paired DNA-RNA hybrids can lead to significant DNA unwinding, upon which Cas9 will cleave both DNA strands (Figure 1, nuclease activation and cleavage). This explains the high sequence specificity in the PAM-proximal region observed for CRISPR-mediated gene editing [49], as well as the recent finding that off-targeted Cas9 binding through the beginning of the PAM-proximal sequence only rarely leads to off-targeted enzymatic activity* in vivo* [43]. Because unwinding the DNA duplex across the first-10~12-nt preconfigured seed sequence might be the critical thermodynamic hurdle to establish stable Cas9 interaction with DNA and subsequent cleavages, a high degree of sequence fidelity in this seed sequence might be both sufficient and necessary via strand replacement to trigger Cas9 conformational changes and remodeling of the active sites. In theory, based on this model, the mismatch of a DNA-gRNA hybrid occurring closest to the PAM sequence should be the least tolerated and is indeed the least common among observed off-targeted bindings [43]. Further thermodynamic modeling based on this model and structural information will likely improve both the efficiency and specificity of CRISPR applications.

## 5. On-Target and Off-Target Considerations

Similar to most other engineering applications, specificity and efficiency are the main factors ensuring a rational CRISPR-experiment design. In subsequent discussions, specificity is defined as the probability that Cas9 will target the designed locus compared to other undesirable loci (off-target effects). Efficiency is defined as the probability that the locus of interest will be modified by Cas9 nuclease in the context of a pool of available target chromosomes from the cell population. In a word, vigorous CRISPR design tends to minimize the off-target effect and maximize the on-target effect of the designer nuclease to achieve both high specificity and efficiency.

The 18~20-nt spacer region, designed as the protospacer sequence in the gRNA, is the main determinant for both off-target and on-target effects of CRISPR experiments. Together with a given adjacent PAM sequence, a gRNA with a 20-nt protospacer region can achieve, in theory, unique sequence recognition in a random sequence space of roughly 17 TB (tera-base pairs) if a perfectly base-paired match is required for targeting. While this theoretical upper limit of resolution exceeds the size of most eukaryotic genomes, the practical specificity of Cas9 was found to be magnitudes lower than the theoretical expectation. It was discovered that the “NGG” PAM sequence requirement of spCas9 was not absolutely necessary since a “NAG” PAM is frequently tolerated with a lower efficiency [12]. The scientific community also quickly realized, since the onset of development of CRISPR genome engineering, that mismatches between the protospacer and targeting DNA are tolerated at a surprisingly high frequency, especially for the 5′ sequence of the protospacer [41, 42, 44, 50]. Further elucidation of Cas9 enzymology revealed that this bias might be due to the unidirectional (3′ to 5′) DNA double-strand melting coupled with DNA-RNA duplex formation upon PAM recognition by Cas9 nuclease. While the gross 3′ to 5′ relaxation gradient of the base-pairing requirement of Cas9 targeting generally holds true, it was found that sometimes sequences with mismatches to the 12-nt seed sequence in the gRNA spacer can be efficiently targeted [39, 41, 42]. This suggests that proper base pairing with the gRNA seed sequence alone does not guarantee specificity. Furthermore, targeting efficiency at some off-target sites could be even higher than the desired locus with perfectly matched spacer-protospacer sequences [39, 41, 42]. This phenomenon might be caused by additional factors beyond the RNA-based sequence recognition used by Cas9 nucleases.

Compared to the considerable knowledge for the basis of Cas9 off-target effects, relatively little is known about how to design a gRNA to make the desired targeting event more efficient. Multiple factors determine the success of any given CRISPR experiments, such as the quantity of Cas9 proteins and gRNA, chromatin accessibility of the targeting loci, and cellular response to CRISPR-induced DNA lesions. Most of these issues are beyond experimental controls when a CRISPR experiment is designed. A few recent studies [51–53] attempted to debug the sequence preference of effective gRNA by retrieving the successful targeting gRNA sequences in a large, randomly selected gRNA pool. This statistical approach is limited by current capability to generate a gRNA pool with sufficient diversity and the difficulties avoiding artificial bias when selecting the efficiently targeted cell pools. Nevertheless, a few statistically significant rules have been revealed by these pioneering studies on common traits of efficient gRNA for spCas9. (a) Guanine (G) is strongly favored at the 3′ position most proximal to the PAM sequence (especially the −1 position). This preference might be due to Cas9 loading [51]. (b) A series of thymine (T) is disfavored at the four positions (−1 to −4) closest to the PAM, which might be related to the fact that RNA polymerase III recognizes a series of uracil (U) as a pausing/termination signal [54], causing a lower level of gRNA expression [51]. (c) Cytosine (C) is preferred at the DNA cleavage site (−3 position). (d) In the PAM region, the +1 position favors C while disfavoring T [52]. (e) The CRISPR activity correlates with gRNA stability, which can be influenced by the nucleotide composition of the spacer: G-rich spacers are more stable especially when comparing with A-rich ones [55].

The emerging gRNA design rationale discussed above was continuously incorporated into available bioinformatics toolboxes as weight matrices for calculating the off-target or on-target scores for any gRNA [52, 55–59]. Although these scores are informative in facilitating the experimental design process, potential CRISPR users should be cautious about interpreting gRNA ranking based on these scores, since it does not necessarily indicate superior specificity and efficiency.

## 6. CRISPR/Cas9 Delivery Methods

As an efficient, RNA-guided, specific gene-modification tool, CRISPR was widely used in many experimental settings to achieve desired mutations. However, the delivery of the required Cas9 protein and gRNA is a long-standing challenge [60]. Three methods of CRISPR delivery, including plasmids, viruses, and ribonucleoproteins (RNPs), were shown to successfully introduce Cas9 and gRNA into target cells and accomplish guided gene editing [11, 49, 61]. With their various merits and limitations, these three delivery methods offer researchers an opportunity to optimize their gene-editing procedures based on various experimental needs.

### 6.1. Delivery Using Plasmid Vectors

Delivery using the plasmid vector system is the conventional and most popular method for CRISPR introduction. It has the main advantage of being simple to make* in vitro*. In order to introduce a functional CRISPR system into target cells, cells need to be transfected with plasmids encoding the Cas9 protein, crRNA, and tracrRNA while simultaneously using electroporation or cationic lipid-mediated delivery to achieve assembly of the CRISPR complex in cells [11].

The plasmid system procedure was continually simplified, and its application range expanded to* in vivo* animal studies. Instead of cloning three different plasmids encoding three different components, researchers showed that plasmid encoding gRNA, a fusion transcript of crRNA and tracrRNA, is sufficient for Cas9 binding and DNA target-site recognition [10]. Recently, plasmids encoding both Cas9 and gRNA became commercially available. Therefore, transfection of a single plasmid is the sole requirement for a CRISPR experiment. Multiplex edition of target loci can be accomplished through simultaneous introduction of multiple gRNA species by a single plasmid or by cotransfection of multiple plasmids [13]. Plasmid delivery was also applied in a tissue-specific CRISPR application in murine liver [60, 62]. Through hydrodynamic tail-vein injection, plasmids were efficiently delivered to ~20% of hepatocytes for transient expression. This study demonstrated successful gene editing with limited efficiency* in vivo* through direct plasmid delivery.

However, compared to successful delivery* in vitro*, the plasmid delivery system still faces significant challenges for* in vivo* applications, such as low delivery efficiency and frequent epigenetic silencing on episomal DNA [63]. Conversely, plasmid delivery offers the dual possibility of both long-term and transient CRISPR delivery* in vitro*. In a small proportion of transfected cells, random but stable integration of all or part of plasmid DNA into the host genome occurs. This is possibly due to low levels of spontaneous DNA damage, which in turn provide continuous Cas9 and gRNA sources [11, 49, 61, 64]. When this feature is not desirable, delivered plasmids usually become diluted and gradually lost over a few cell cycles. This limited time window of genome engineering is critical for obtaining genetic homogenous cell populations for downstream functional studies.

### 6.2. Delivery Using Lenti-, Adeno-, and Adeno-Associated Viral Vectors

The plasmid system introduces CRISPR into established cell lines efficiently. However, to expand CRISPR's application range, viral vectors are used to deliver CRISPR into primary cells or cells refractory to plasmid transfection. Lentiviral vectors stably integrate into the host genome, making it the preferred means of delivery if the targeting information needs to be retrieved after functional selection processes [51, 65–67]. It is now feasible to carry out genome-wide, CRISPR-based, functional genomic screens by delivering complex pools of CRISPR reagents into a relevant cell type via lentiviral packaging. One significant limitation of lentiviral-based delivery is that the random integration of a viral genome may cause unwanted insertional mutagenesis at undesired host loci. Use of nonintegrating viral vectors (NIVVs), including adenoviral vectors and adeno-associated vectors, can efficiently circumvent this problem because they do not incorporate viral DNA into the host genome [11, 60]. Moreover, viral DNA dilutes during mitosis due to the lack of a replication signal [60]. Among NIVVs, adenoviral and adeno-associated vectors are both potentially suitable CRISPR delivery candidates because of their episomal nature, large cloning capacity, high-titers, capability of long-term* in vivo* expression, and ability to transduce many cell lines [39, 49, 61, 62].

While a viral vector encompassing Cas9 and gRNA expression cassettes can be produced at high-titers, the negative correlation of packaging efficiency versus vector size also poses challenges for single-vector delivery of both Cas9 protein and gRNA. Successful gene editing was achieved using adenovirus-delivered CRISPR in multiple mammalian cells. Using different gRNA and Cas9 virus concentrations, researchers showed that the editing efficiency is dosage dependent [10, 61]. Besides transfection of stable cell lines, adenoviral vector-mediated CRISPR delivery can also be applied* in vivo*. Through tail-vein injection, adenoviruses carrying Cas9 and gRNA expression cassettes can be introduced into murine liver. Resulting Cas9-mediated gene editing is stable even after extensive regeneration of liver tissue [13, 68]. Compared to hydrodynamic tail-vein injection of plasmids, tail-vein injection of adenoviruses achieved 5- to 8-fold greater editing frequency [69]. This high efficiency makes virus-delivered CRISPR an attractive option for* in vivo* genome modification. However, systematic delivery using the adenovirus vector* in vivo* could induce immune responses that eliminate infected cells and eventually impair CRISPR genome-editing efficiency. In one recent study using adenoviral vector delivery, the transduction rate of liver cells drops from 80.8% one day after injection to 1.4% fourteen days after injection. This is most likely due to the immune response of the host, including elevated expression of inflammatory cytokines [31, 69]. In contrast, the adeno-associated virus (AAV) induces a mild immune response* in vivo* and can provide long-term expression in nondividing cells. The recent study using* Staphylococcus aureus* Cas9 (SaCas9) solved the viral packaging limit problem for spCas9, making the AAV-mediated delivery an ideal method for* in vivo* genome editing [31].

### 6.3. Delivery Using Cas9-gRNA Ribonucleoproteins (RNPs)

In addition to plasmid vector and viral vector delivery, CRISPR delivery using Cas9-gRNA RNPs is another established method [64]. Both plasmid and viral delivery encountered the problem of high off-target editing rates due to prolonged expression of Cas9 and gRNA in cells. Using direct delivery of RNPs can effectively circumvent this problem. When injected directly into cells, RNPs induce editing at target sites immediately after delivery and degrade rapidly, reducing off-target effects [70, 71]. Additionally, using RNPs avoids the possibility of undesired DNA integration into the genome due to its DNA-free mode of delivery.

Application of RNP delivery led to successful genome editing in multiple human cell lines [64, 72]. The RNP complex can be readily made through incubating* in vitro* purified Cas9 protein with either a single-chain guide RNA (sgRNA) or dual RNA that consists of crRNA and tracrRNA. Under certain circumstances, dual RNA was shown to be more effective than single gRNA [73]. Direct injection of RNP complexes into cells can lead to efficient CRISPR-mediated genome editing with high specificity and low off-target rates compared to plasmid delivery [64]. RNPs are traditionally delivered by direct microinjection in a low-throughput manner. Recently, the feasibility of transfecting CRISPR RNPs into cells efficiently using electroporation was demonstrated [72], as well as using cationic lipid-mediated liposome delivery [74]. Delivery of RNPs into cell-cycle-synchronized cells also yielded a significantly higher rate of editing compared to delivery in nonsynchronized cells. More importantly, researchers can maximize the utilization a particular mode of double-strand break (DSB) repair by delivering RNPs into cells arrested at a particular cell-cycle phase [72]. Continual improvement of RNP delivery makes it a prominent method for not only gene editing in an experimental setting, but also clinical gene therapy development.

## 7. CRISPR Efficiency Test

### 7.1. Test of Indel (Local Point Mutation, Insertion, and Deletion)

**Figure 2:**
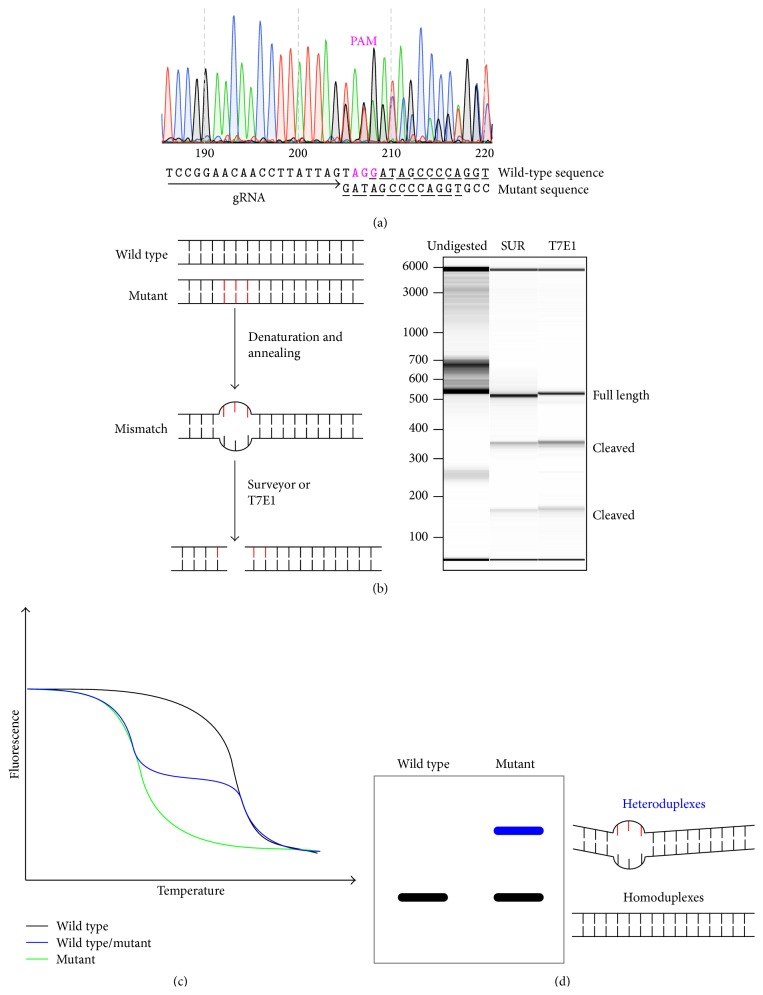
Major methodologies for mutation detection. (a) Sequence decoding from Sanger sequencing. An example of a Sanger sequencing read was shown to illustrate the significant decrease of read quality from the predicted CRISPR cut site (PAM position labeled by magenta). This is due to the inclusion of the mutated DNA (decoded as the bottom sequence) with the wild-type DNA sequence (decoded as the top sequence). Underlined sequence reveals identical nucleotides between the wild-type and mutant sequences, which indicates the major mutation is a 3-nucleotide (TAG) deletion. (b) Recognizing mismatched dsDNA using the single-stranded specific nucleases. Mixed sequences with local sequence polymorphisms (CRISPR-induced indel mutations) form a mismatch when rehybridizing. The result from the mismatch-recognizing nuclease assay is visualized using fragment analysis as a digital nucleic acid size profile. (c) High Resolution Melting Analysis. (d) PAGE electrophoresis of a DNA hybrid.

When assembled with gRNA, Cas9 nuclease cleaves dsDNA and induces DSBs. DSBs can be repaired by either nonhomologous end joining (NHEJ) or homologous recombination (HR). NHEJ is an error-prone process that generates random insertion or deletion (indel) mutations at the DNA rejoining sites. Sanger sequencing is the most accurate way of confirming indel mutations (Figure 2(a)). However, due to the random nature of indels, a wide variety of mutated DNA might be present after a CRISPR-induced NHEJ process. Separating these molecule species using molecular cloning coupled with Sanger sequencing is time-consuming and cost-inefficient [75]. Recent progress in bioinformatics tools (such as TIDE, Tracking of Indels by DEcomposition) enabled successful digital decoding of Sanger sequencing from a mixture of complex indels, generated by a unique CRISPR-targeting event into separate mutant species [76–78]. Although this method is still of limited sensitivity and remains to be validated on a larger scale, Sanger sequencing of a locally amplified, targeted locus offers a quick and reliable readout confirming the efficiency of any given CRISPR experiment. Without sequencing, the separation of DNA with minor differences of length (resulting from some indels) on a Sanger sequencer can be used to quickly access the success of a genome-editing experiment. IDAA (Indel Detection by Amplicon Analysis) was recently developed to fill this niche [79]. Through the use of target-specific primers flanking the target site, the different sizes of amplicons can be detected [79]. Furthermore, several other methods that take advantage of NHEJ-induced indels were developed to efficiently assess the cleaving efficiency of CRISPR through the detection of indel mutations at target loci regardless of DNA length change; these include the Surveyor nuclease assay, the T7 Endonuclease I (T7E1) assay, the High Resolution Melting Analysis (HRMA), and PAGE electrophoresis [80–85].

Surveyor, T7E1, and other nuclease-based mutation detection assays rely on the formation of a locally mismatched heteroduplex DNA, a byproduct of sequence variation caused by NHEJ following the designated nuclease target (Figure 2(b)). If CRISPR-mediated cleavage is successful, indels will be generated at the DSB sites through NHEJ. Heteroduplex DNA can be formed after melting and rehybridizing mutant and wild-type alleles. The mismatch-recognizing enzymes, such as Surveyor and T7E1 nucleases, can detect heteroduplex DNA. Bacteriophage resolvase T7E1 recognizes and cleaves distorted dsDNA undergoing conformational changes [86]. Surveyor nuclease is a single-stranded nuclease that recognizes a nucleotide mismatch induced by indels. It not only cleaves DNA one strand at a time on the 3′ end, but also contains 5′ exonuclease activity [87, 88]. Both enzymes recognize indels and induce DSBs at mismatch sites, resulting in shortened DNA fragments of various sizes. The digested DNA fragments can then be visualized using gel electrophoresis or DNA fragment analysis [82, 88]. However, both enzymes exhibit low levels of random single-stranded nuclease activity, leading to unspecific cleavage. This problem can be partially resolved through addition of Ampligase during the enzyme nuclease reaction [89], which reduces the nonspecific nuclease activity.

HRMA is another tool for indel detection, utilizing the different denaturation profile of heteroduplex DNA compared to that of homoduplex DNA (Figure 2(c)) [90]. If CRISPR-induced indel is present in template DNA, heteroduplex and homoduplex DNA will be formed after melting and rehybridizing mutant and WT alleles. Different duplex species exhibit different denaturation patterns. HRMA records the temperature-dependent denaturation profile of the sample and determines the existence of heteroduplex DNA based on different melting patterns from the sample mixture. Due to its sensitivity, HRMA requires proper optimization of PCR conditions to ensure high specificity of target amplification.

The polyacrylamide gel electrophoresis- (PAGE-) based method was recently proven to be efficient in detecting the presence of heteroduplex DNA (Figure 2(d)) [85]. This method takes advantage of the migration speed difference between heteroduplex and homoduplex DNA during native PAGE. Heteroduplex DNA generally migrates at a much slower rate due to its indel-induced open angle between matched and mismatched DNA strands and therefore can be visualized using PAGE. However, whether the PAGE assay provides sufficient sensitivity across the spectrum of indel mutation variation remains to be verified.

### 7.2. Sensitivity Issues and Reporter

While CRISPR is considered an accurate genome-editing method, the efficiency of CRISPR varies significantly when applied to distinct loci and different cell types. In induced pluripotent stem (iPS) cells and human embryonic stem cells (hESCs), for example, CRISPR-editing efficiency frequently drops below 1% [91, 92]. This low frequency increases demand for more sensitive rare mutation detection methods. Sanger sequencing is the gold standard for determining on-target edition efficiency, yet it is a time- and resource-consuming process. When the mutation rate falls below a given threshold (usually ~1%), routine mutagenesis detection methodologies (Sanger sequencing, nuclease-based heteroduplex cleavage assay, HRMA, and PAGE) are of limited use due to their sensitivity restraints. High-throughput sequencing was developed for accurate measurement of rare indels that happen at a frequency of 0.01–1%. However, because this method is considerably more sensitive than traditional methods (such as mismatch-recognizing enzymes) the false-positive frequency is also elevated [75].

Single molecule real-time (SMRT) DNA sequencing was developed as a unique high-throughput sequencing platform [93]. It has the advantage of both high sensitivity and long reading length. A regular PCR amplified region of interest is ligated with SMRT adaptors to create a single molecule SMRTbell template to generate sequence reads. This method not only examines the existence of an editing event, but also quantifies the frequency of editing through either NHEJ or HR. With an average sequencing length of 3 kb and up to 15 kb, SMRT sequencing provides a reliable method for assessing both on-target and off-target rare editing effects. Similarly, other high-throughput sequencing platforms can be applied to quantitate indels in the targeted amplicon.

To further assess CRISPR-editing efficiency using accurate quantification for very rare editing events, digital droplet PCR (ddPCR) can be applied to CRISPR-edited genome testing [94]. Depending on the assay format, ddPCR assay has theoretical mutation detection limits in the range of 0.01~0.001%. To achieve individual assessment of the edited genome, sample DNA is partitioned into small droplets through emulsion. One set of primers flanking the region of interest and two competitive fluorescence-tagged probes targeting wild-type and mutant sequences, respectively, are included in the reaction. An individual PCR reaction is carried out in each droplet, and fluorescence signals from each droplet are subsequently recorded. The wild-type and mutant sequences are differentiated, and the frequency of editing can be calculated based on the number of droplets with different fluorescence signals [91]. This method allows extremely sensitive detection of rare mutations as well as accurate quantification of CRISPR-editing efficiency. Novel ddPCR application was explored in other studies, including differentiating wild type and mutants based on the size of amplicons using the nonspecific, double-strand DNA binding dye EvaGreen (EG) [95].

Besides quantifying CRISPR-induced indels, live reporters based on HR can be used to visualize CRISPR activity. Typically, a reporter plasmid vector can be designed to include the identical target-site sequence as the targeting locus. The CRISPR target is flanked by two separate halves of a fluorescent protein reporter, with a stretch of an identical sequence included in both halves. Thus, this reporter is inactive since the fluorescent protein gene is interrupted by the inserted sequence. CRISPR components and the reporter plasmid are cotransfected. Efficient gRNA loads Cas9 to cleave both the chromosomal targeting locus and the episomal reporter-targeting site. In the reporter, the DSB will be repaired through HR between the two halves of the fluorescent protein, thus rendering a fully functional fluorescent protein. Hence, the “on” status of the reporter plasmid, exhibited by the gain of the cellular fluorescence signal, can give a real-time readout of CRISPR efficiency in live cells independent of additional molecular assays.

## 8. Selection of Mutant Clones 

Pure clonal isolation from a single progenitor cell is a critical step in the genetic and functional characterization of mutations achieved by the CRISPR/Cas9 system. While it is usually the most laborious and time-consuming step in CRISPR-based genome engineering using cell models, generating clonal mutant cell lines is absolutely required to draw any solid conclusions correlating a given mutation and cellular behavior. Each single cell, upon the introduction of activated Cas9 nuclease, is an independent unit that undergoes stochastic genetic changes dependent on both the nuclease-induced DNA lesion and the subsequent cellular DNA-repair response. In the case of transient introduction of CRISPR agents, it is desirable to establish clonogenic cultures by the conclusion of CRISPR action. In the stem cell research field, a clonogenic culture is frequently confused with the sphere generating culture, such as formation of embryonic bodies from ES cells or neurospheres from neuronal stem cells [96]. While these sphere-forming assays are frequently used to estimate the capability of stem cells to self-renew and differentiate, the individual spheres formed in standard stem cell culture conditions do not necessarily rise from single cells [97], since sphere aggregation and fusion were frequently found even at low seeding densities [98–100]. The requirement of clonogenity after CRISPR action usually calls for more rigorous culture conditions to ensure proper clonal separation of distinct isogeneic pools.

There are multiple methods to achieve clonogenity. To prevent sphere fusion, single cells can be encapsulated into a semisolid matrix to form embedded sphere cultures [101]. This approach greatly improves the clonogenity of the spheres generated and offers greater advantage when cell proliferation is strictly dependent on high cell density in the culture [98]. However, single-cell encapsulation usually requires specific microfluidics devices [102]. Furthermore, maintaining capsule integrity and retrieving encapsulated cells remain challenging. Aside from cell encapsulation, cells grown in semisolid media, such as those containing methylcellulose or soft agar, are less likely to migrate [103]. When seeded at low density, single cells in semisolid media can grow into individual colonies over time. Manual or robotic selection of these colonies can subsequently establish isogenic clones. The traditional labor-intensive ways to establish cultures from single cells include cloning rings, serial dilution and plating, and fluorescent-based single-cell sorting [104, 105]. Regardless of the methodology, establishing and maintaining a large number of isogenic cell clones are costly and labor-intensive. For most genome-engineering experiments, the optimally desired approach should minimize the number of isogenic cell clones needed to achieve the desired genetic modification. In the following sections, the factors to achieve this goal will be discussed.

### 8.1. Overall Strategy, NHEJ or HR

DSBs in the eukaryote genome can be repaired mainly by two different mechanisms: NHEJ or HR. The NHEJ repair mechanism joins broken chromosomal ends directly without the guidance of a homologous sequence. Because it lacks a reference template, this repair pathway is usually error-prone due to local DNA sequence alterations at the repaired junction (the so-called indels) [106]. In contrast, the HR repair mechanism is aided by using a homologous sequence as the repair template. This homologous sequence can be a sister chromatid duplicated during the synthesis (S) phase of cell cycle, the homologous chromosome in diploid cells, or foreign DNA introduced bearing regions of sequence homology with the targeted locus. Due to the flexibility of donor choice in HR repair, a given locus with desirable features (such as restriction enzyme recognition sites, protein fusion tags, antibiotic selection markers, or recombination sites) can be engineered by incorporating these features with a piece of introduced homologous DNA. Either plasmid construct or synthesized DNA oligos can be used as the donor template [40]. A plasmid donor can be used when long insertions need to be introduced [107, 108]. For small insertions or deletions, single-stranded DNA containing 80 bp homologous arms at 5′ and 3′ ends is preferred [107]. This method is similar to traditional HR-based gene targeting. However, since the introduced DSBs occur in the chromosomal DNA instead of epichromosomal DNA, the HR efficiency is usually several orders of magnitude higher than traditional HR triggered by breaking the foreign donor [3, 108–111].

While the choice of DNA-repair pathways is largely beyond experimental control, the cell-cycle phase upon which DSB occurs plays an important role in repair mechanism determination. In general, HR takes place in the synthesis (S) and the premitotic (G2) phases when there are sister chromatids available [112]. NHEJ is the predominant repair mechanism in the growth 1 (G1) and the mitotic (M) phases [113]. Although this general guideline holds true in most cases, precautions are warranted for any particular cell type for its capability on HR- or NHEJ-based DNA-repair pathways.

Regardless of the preferred DNA-repair mechanisms to get a particular or a range of desired mutations, similar clonogenic selection processes are needed. Since HR usually happens at a lower frequency than NHEJ for most cell types, it is an efficient strategy to include a selection marker on the donor construct so that successfully engineered cells can be easily traced by fluorescence or drug resistance. The marker is integrated onto the targeted loci. In some cases this feature is not ideal for downstream functional analysis, even when the majority of the selection markers can be subsequently excised by recombinases.

**Figure 3:**
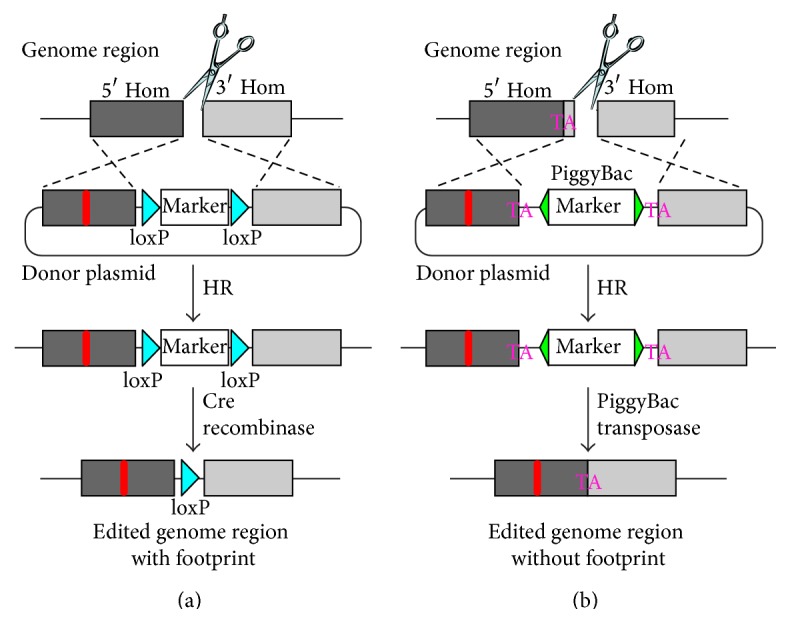
The comparison of seamless genome editing with traditional HR-based marker selection. (a) Traditional HR. (b) Seamless genome editing. Homology arms (dark grey and light grey boxes) bearing the desired mutation (red bar) are used to flank an excisable selection marker cassette. This is achieved by using the tandem loxP sites as in (a) and a PiggyBac transposon as in (b). Successful HR will insert the selection marker cassette into the genome (middle panels). Removing the loxP cassette with Cre recombinase will leave one loxP site at the locus of interest (blue triangle) in (a). The remobilization of the PiggyBac transposon will only leave a “TA” dinucleotide in (b), which initially can be found in the locus of interest, or can be tolerated without any undesired changes to the protein sequence.

A few seamless genome-engineering applications emerged in the last few years to overcome this hurdle. This elegant approach aims to introduce only the desired genetic modification without leaving additional footprints at the engineered loci (including indels at the CRISPR cut sites, any selection markers, or short residual recombination sites after marker excision) (Figure 3) [24, 114, 115]. To facilitate clonal selection, a selection marker is included in the DNA donor similar to traditional HR. However, instead of using a recombinase to induce flanking recombination sites around the marker, which would leave behind at least one recombination site (Figure 3(a)), an optimized PiggyBac transposon is used for all exogenous sequences between the homology arms. Only a “TA” dinucleotide sequence is left on each side flanking the exiting PiggyBac (Figure 3(b)). To make this truly seamless, the left and right homology sequences start with a “TA” motif, which is abundant in most genomic loci. If there is no endogenous “TA” around the intended mutation, it is usually feasible to introduce one without changing the translated protein sequence in exons or make this change in mutation-tolerating introns. A negative selection marker is usually included in the PiggyBac cassette in the designed DNA donor to facilitate screening the loss of the PiggyBac cassette by the transposase. This method holds great promise for CRISPR-mediated site-specific gene therapy, since avoiding any additional sequence modification is highly desirable.

Regardless of the choice of methods, clonogenic clone isolation and identification are labor-intensive. To design the most effective screening strategy, it is crucial to realistically estimate the chance of obtaining the desired mutant cells in the pool undergoing CRISPR-mediated genome engineering. A critical factor is the efficiency of CRISPR targeting the locus of interest, which can be tested by a small-scale pilot experiment using the mutation detection methodologies discussed in the previous section. Depending on the mode of DNA-repair pathway chosen, further consideration can be made regarding whether it is feasible to first reduce the size of the cell pool by selection to enrich the targeted cells before clonal assay. Isolating cells positive for the HR-mediated live-cell cleavage reporter could enrich NHEJ-mediated indel mutations [116]. Although these are achieved by different mechanism of DNA repair, the reporter assay may indicate the subpopulation of cells where CRISPR is more active. Similarly, if the desired mutation was introduced using HR repair, inclusion of the selection marker in the DNA donor could be an efficient way to reduce the size of clonal selection pool. Frequently, the intended mutation might be predicted with high confidence to cause a specific cellular phenotype in the target-cell type. If the specific cellular phenotype can reliably be used for selection, target-cell enrichment can be achieved by applying this selection pressure [117]. Without highly efficient CRISPR reagents, a target selection scheme is required to move the mutation frequency above 0.1%, in order to make clonal single-cell selection feasible.

In cases of low mutagenesis frequency and no suitable selection strategy available for mutant enrichment, a random cell partition scheme named sib-selection can be employed to facilitate enrichment of the desired mutation before clonal isolation [91, 118]. Sib-selection is based on precise measurements of mutation frequencies in pools of cells even when the rate is extremely low. The ddPCR method was used for this purpose to gain a reliable quantitative mutation rate. When a pool of cell mixtures with a rare mutant is sequentially partitioned randomly into smaller pools (such as different wells in a 96-well plate), the mutation rate in one or a few small pools will increase significantly due to the overall significant decrease of cells in a pool following a Poisson distribution. The capability to locate these enriched wells using a quantitative mutation measurement can facilitate serial pool partition and mutant identification, until the rate of desired mutants surpasses the practical threshold for clonal identification. Although a powerful and quick way to enrich mutation, sib-selection is not a clonogenic process* per se*. Thus subsequent clonal mutant strain identification is needed to isolate the intended mutant cell.

### 8.2. Estimation of Off-Target Mutations in Isolated Cell Clones

Acquiring pure cell populations with the desired genetic modifications should not be considered as the final step before using these cell models for functional studies. No matter how carefully the experiment was designed, it is likely that some off-target modifications were introduced into the cell pool by CRISPR. If any of these are carried on into the final selected clones, these additional genetic modifications might complicate further functional analysis.

Whole genome sequencing of the isolated cell clones remains the most rigorous standard to estimate the off-target lesions [119–121]. It remains expensive, especially for human cells, since the complete genome requires a significant sequencing depth to detect the occurrence of low frequency indels. While its costs prohibit routine use to examine all isolated cell clones in a typical lab, a reasonable approximation can usually be made by targeted sequencing of predicted off-target sites. This can be done in a low-throughput manner using PCR and Sanger sequencing of a number of individual predicted off-target sites with significant targeting probability. Alternatively, multiplexed next-generation targeted sequencing can be achieved by covering a large number of off-target sites simultaneously from multiple single-cell clones with significant sequencing depth [46, 122]. In the case of targeted sequencing, the choice of examined genomic region becomes critical. While various* in silico* platforms give a rough estimate of potential off-target sites, recent advances on genome-wide breakpoint sequencing technology (such as CHIP-Seq [43, 122], Digenome-seq [123] and GUIDE-seq [124], and genome-wide translocation sequencing [125]) offer a more realistic range of potential off-target sites in any given genome. While these platforms collectively can aid targeted genome sequencing of the engineered cells, precautions are still warranted since off-target CRISPR targeting can be influenced by the different cell types used and minor differences of genome sequence [126]. Some additional practical precautions should be taken into consideration, especially when the undesirable off-target lesions are not sufficiently characterized or hard to avoid.

### 8.3. Correlating Phenotype and Genotype Controls

When a certain phenotype is displayed after CRISPR-mediated editing in the clonogenically isolated mutant cells, the phenotype is not necessarily caused by the intended target due to the possibility of poorly characterized off-target lesions. The genotype/phenotype association can be strengthened by verification using additional clonogenic clones carrying independent mutations generated by different CRISPR agents targeting the same locus. Because identical off-target lesions might be generated by the same gRNA, it is not possible to strictly exclude this possibility by relying on additional clones generated by a single gRNA. Therefore, additional gRNA is desired to target the same region of interest to achieve the identical phenotypic outcome. With limited overlapping of off-target sites, multiple gRNA designs ensure that any shared phenotype exhibited after editing using all gRNA correlates with the genotype of interest with high confidence. Aside from establishing proper controls for CRISPR targeting, genetic rescue is considered the gold standard to formally establish the causal relationship between phenotype and genotype. For loss of function mutations, introducing the intact target genes or gene products into the engineered cells should serve the purpose. Introducing the gene of interest back into the endogenous engineered locus is readily achievable by CRISPR [127–129] and is preferable, since the rescue genetic material is under endogenous transcriptional control. In the case of gain-of-function mutations where genetic rescue is difficult to achieve, pharmaceutical genetic approaches are useful in functional validations. Fine-tuning the functionality of a given target or relevant pathways using well-characterized specific drugs could provide independently supported evidence.

## 9. A Much Brighter Future for Stem Cell Models

The accumulation of large-scale human genome-sequencing efforts in the past few years greatly accelerated genetic discovery by linking genetic variations discovered in human populations or disease-associated somatic tissue to a disease state. Stem cell models, on the other hand, are traditionally extremely powerful in establishing the mechanistic linkage between genotype and phenotype. The recent explosion of applications of CRISPR/Cas9 genome-editing techniques now establishes the causal relationship between genotype and cellular behaviors with great flexibility and efficiency. While our current review can grasp neither the full extent nor the rapid evolution of these applications, a few prominent examples are highlighted below to demonstrate the range and depth of these applications.

One of the earliest successful applications of CRISPR in stem cell research was to correct the CTCF mutation in cultured intestinal stem cells from cystic fibrosis (CF) patients [130]. Besides fixing local sequence errors, CRISPR was recently used to correct a chromosomal structural abnormality (a chromosomal inversion over a several-hundred-kilo-base-pair) associated with Hemophilia A [131]. Using stem cell models (especially patient-derived iPSCs), CRISPR was used to correct more than a dozen disease-associated genetic lesions across a wide spectrum [115, 130–143], including metabolic disorders, immunological deficiencies, and neuromuscular disorders. These genetically corrected, patient-derived stem cells might be the critical vehicle for future cell and gene therapies, with further improvement on its safety.

Regardless of its therapeutic potential, CRISPR is an invaluable tool in establishing the causal relationship between genes and stem cell behavior. Clevers group recently modeled the occurrence of the 4 most frequent mutations identified in human colorectal cancer within the context of a human intestinal stem cell organoid culture. This analysis enabled them to pinpoint the driver mutations causing extensive aneuploidy within this cancer stem cell model [117]. CRISPR also helped to pinpoint a specific single-nucleotide polymorphism (SNP) in the human FTO locus as the critical effector for obesity [144]. Previous genome-wide association studies indicated the FTO region harbors the strongest genetic association with obesity, while no mechanistic association could be drawn. A SNP in the FTO locus was further nailed down as the obesity-causing variant. Modeling the conversion of this one nucleotide using CRISPR in the context of isogenic, patient-derived preadipocytes provided the critical link between this single-nucleotide substitution and distinct adipocyte differentiation programs: thermogenic beige adipocytes versus fat-storing white adipocytes. This stem cell model, combined with the power of CRISPR-mediated genome editing to change one particular nucleotide in the human genome, helped resolve one of the longest standing mysteries in human genetics. Thus, we are extremely enthusiastic for a much brighter future for making and using stem cell models for similar mechanistic studies.

## Figures and Tables

**Table 1:** Orthogonal type II Cas9 and their optimal PAM preference.

Bacteria	PAM	CRISPR type	Reference
*S. thermophilus* ^*∗∗*^	NNAGAAW (CRISPR1)	IIA	[28, 29]
*N. meningitidis*	NNNNGATT NNNNGCTT	IIC	[28, 47, 145]
*T. denticola*	NAAAAN	IIA	[29]
*S. mutans*	NGG	IIA	[47]
*L. innocua*	NGG	IIA	[47]
*L. buchneri*	NAAAAN	IIA	[47]
*C. jejuni*	NNNNACA	IIC	[47]
*P. multocida*	GNNNCNNA	IIC	[47]
*S. aureus* ^*∗∗*^	NNGRRT	IIA	[31]
*N. cinerea*	GAT^*∗*^	IIC	[31]
*C. lari*	GGG^*∗*^	IIC	[31]
*P. lavamentivorans*	CAT^*∗*^	IIC	[31]
*C. diphtheriae*	GG^*∗*^	IIC	[31]
*S. pasteurianus*	GTGA^*∗*^	IIA	[31]
*S. pyogenes*	NGG (NAG as minor)	IIA	[10]

*S. pyogenes* (D1135E)	NGG (does not recognize NAG)	IIA	[35]
*S. pyogenes* VQR (D1135V/R1335Q/T1337R)	NGAN NGCG	IIA	[35]
*S. pyogenes *EQR (D1135E/R1335Q/T1337R)	NGAG	IIA	[35]

^*∗*^Putative PAM; ^*∗∗*^significantly smaller than spCas9. Bottom rows are engineered spCas9 proteins with different PAM preferences.

## Abbreviations and Acronyms

ZFN: Zinc Finger Nucleases
TALEN: TALE domains in transcription activator-like effector nucleases
CRISPR/Cas: Clustered regularly interspaced palindromic repeats/CRISPR-associated
tracrRNA: Transactivating CRISPR RNA
crRNA: CRISPR repeat RNA
PAM: Protospacer adjacent motif
RNP: Ribonucleoprotein
gRNA: Guide RNA
dsDNA: Double-stranded DNA
DSB: Double-strand break
NHEJ: Nonhomologous end joining
HR: Homologous recombination
PAGE: Polyacrylamide gel electrophoresis
HRMA: High Resolution Melting Analysis
CHIP-Seq: Chromatin Immunoprecipitation Sequencing.
